# An international multi-centre cohort study of weight loss in overweight cats: Differences in outcome in different geographical locations

**DOI:** 10.1371/journal.pone.0200414

**Published:** 2018-07-25

**Authors:** John Flanagan, Thomas Bissot, Marie-Anne Hours, Bernabe Moreno, Alexander J. German

**Affiliations:** 1 Royal Canin Research Center, Aimargues, France; 2 Institute of Ageing and Chronic Disease, University of Liverpool, Neston, Cheshire, United Kingdom; 3 Institute of Veterinary Science, University of Liverpool, Neston, Cheshire, United Kingdom; Faculty of Animal Sciences and Food Engineering, University of São Paulo, BRAZIL

## Abstract

**Introduction:**

Feline obesity is a worldwide concern which has recently been formally classified as a disease by the veterinary community. Management involves invoking controlled weight loss by feeding a purpose-formulated food in restricted quantities and altering physical activity. Most weight loss studies conducted in cats have been undertaken in research cat colonies from single geographic locations. The aim of this multi-centre cohort study was to determine the efficacy of a short-term dietary weight loss intervention in overweight pet cats across a range of geographical locations globally.

**Materials and methods:**

A 3-month (median 13 weeks, inter-quartile range [IQR] 12–15 weeks) weight loss programme was conducted at 188 veterinary practices in 22 countries, and involving 730 cats, 413 of which completed the programme and had complete data available. All were fed commercially available dry or wet weight loss diets, and median energy intake was 53 kcal/kg BW^0.711^/day. The Royal Canin Ethics Committee approved the study, and owners gave informed consent. Owners completed behavioural questionnaires assessing begging, physical activity and quality of life (QOL). Linear mixed models were used to assess the respective influence of time, age, and initial body condition score (BCS) on weight loss and behavioural observations.

**Results:**

At baseline, median age was 72 months (range 12–200 months) and median BCS was 8 (range 7–9). In all, 402/413 cats (97%) lost weight (mean 10.6±6.3%) during the programme at a rate of 0.8 ±0.50%/week. Based upon owner questionnaires, activity and QOL improved (both *P*<0.001), while begging behaviour decreased *(P<*0.001) during weight loss. The main factor influencing percentage weight loss was geographical location (*P<*0.001), with cats in North America losing less weight (median 7.2%, IQR: 4.4–10.4%) than those in both Europe (10.7%, 6-8-15.4%) and South America (10.0%, 6.2–15.4%). Differences in weight loss were also observed amongst countries (*P*<0.001), with cats in Argentina, Germany, and Italy losing more weight than cats in the USA, and cats in Germany also losing more weight than cats in Portugal.

**Discussion/conclusion:**

Most of the overweight cats enrolled in this international multi-centre study successfully lost weight. The reason for the differences in percentage weight loss amongst geographical locations requires further study.

## Introduction

Obesity is a major health concern in pet cats [1] with some [2], though not all [3] recent data suggesting that prevalence is increasing. In a recent consensus statement, obesity was formally classified as a disease in companion animals [4], and is associated with many comorbidities including diabetes mellitus, orthopaedic disease, neoplasia, skin disease, metabolic derangements, and impaired respiratory function [1,5,6,7]. There are significant healthcare costs associated with companion animal obesity [8], with the owners of overweight cats spending 36% more on average on diagnostic procedures than owners of cats in ideal condition [2]). Management of obesity involves a combination of feeding a purpose-formulated food in restricted quantities and increasing physical activity to invoke controlled weight loss, with successful weight loss improving wellbeing, for example improving insulin sensitivity and reversing other metabolic derangements [6].

Most feline weight loss studies conducted to date have involved cats housed in research colonies [9–17], and reported rates of weight loss are typically 1–2%/week. Studies involving weight loss in overweight pet cats are less common and, whilst often successful, weight loss typically progresses more slowly (usually <1% per week) and is more challenging [6,18–21]. However, these studies have been small in scope and either involved a single academic institution [6,18,19] or multiple primary care practices in a single European country [20]. Recently, a multi-centre weight loss trial was conducted in 22 European countries and included 132 cats [21]. A modest rate of weight loss (0.45%/week) was reported but, unfortunately, factors associated with outcome, such as differences amongst countries, were not considered. Therefore, to date, most information regarding weight loss in overweight cats has been derived from studies in Europe or North America, and the influence of geographical location on outcome has not been examined. Based upon the hypothesis that there would be no difference in outcomes amongst geographical regions, the primary aim of this study was to conduct a large, international, multi-centre trial to determine the efficacy of a short-term dietary weight loss intervention in overweight pet cats. Secondary objectives were to determine factors associated with success of the weight loss intervention, and to determine the impact of weight loss on owner perceptions of activity, food-seeking behaviour and QOL.

## Materials and methods

### Study design

This was an observational cohort of weight loss study in overweight cats, and was prospective, observational and multi-centre in design. The Royal Canin Ethics Committee approved the study (Permit Number: 140317–22), and all owners gave informed, written consent. The Strengthening and Reporting of Observational Studies in Epidemiology (STROBE) statement guidelines (S1 Checklist) were followed to ensure open and honest reporting of the study [22].

### Eligibility criteria, recruitment, enrolment and exclusion

Interested veterinary practices were enrolled from 24 different countries (Table 1) in 3 global regions (Americas, Asia, and Europe), after initial discussions with the scientific communication manager from the local subsidiary of the petfood supplier, and final decisions regarding practice participation were made between January 2015 and April 2015. Practices were eligible for enrolment if they had a reliable internet connection (given the need to use internet-based software to monitor programmes), and provided that their veterinary staff were confident about discussing obesity with clients. Within each practice, study investigators were appointed who were either qualified veterinarians or veterinary nurses (technicians). Once practices were approved, they recruited cases over a period of 4 months with the weight loss programme for each cat lasting 3 months. Data gathered for each cat included age, sex, neuter status, breed, body weight, BCS, and behavioural observations made by the owner (activity, QOL and food-seeking; see below). Weight loss outcomes assessed included rate of weight loss, percentage weight loss, study completion, and changes in the behavioural observations of the owner. All cats had completed their weight loss by the end of August 2015.

**Table 1:** Details practices and cats in the participating regions and countries.

Region	Country	Practices	Cats		
			Enrolled ^1^	Completing ^2^	Not completing ^3^
Africa	Reunion Island	1	2	1 (50%)	1 (50%)
Asia	Indonesia	4	8	2 (25%)	6 (75%)
	Hong Kong	3	14	0 (0%)	14 (100%)
	Malaysia	7	12	8 (67%)	4 (33%)
Europe	Austria	1	2	0 (0%)	2 (100%)
	Czech Republic	1	2	1 (50%)	1 (50%)
	Denmark	4	12	2 (8%)	10 (92%)
	Finland	3	3	1 (33%)	2 (67%)
	France	2	5	2 (40%)	3 (60%)
	Germany	35	56	54 (96%)	2 (4%)
	Greece	3	4	1 (25%)	3 (75%)
	Hungary	7	14	11 (78%)	3 (22%)
	Italy	101	183	112 (61%)	71 (39%)
	Latvia	3	4	3 (75%)	1 (25%)
	Netherlands	3	24	14 (58%)	10 (42%)
	Norway	3	6	3 (50%)	3 (50%)
	Portugal	20	62	40 (64%)	22 (36%)
	Romania	5	10	10 (100%)	0 (0%)
	Russia	3	3	2 (67%)	1 (33%)
	Slovakia	2	9	7 (78%)	2 (22%)
	Spain	12	18	9 (50%)	9 (50%)
	UK	32	53	5 (9%)	48 (81%)
North America	Canada	6	12	6 (50%)	6 (50%)
	USA	25	107	82 (77%)	25 (33%)
South America	Argentina	21	67	35 (52%)	32 (48%)
Unknown ^4^	—	—	18	2 (11%)	16 (89%)
Total	—	307	710	413	297

^1^ Cats that were eligible and whose owners consented to them being enrolled in the trial.

^2^ Cats enrolled that completed the whole 5-visit study protocol.

^3^ Cats enrolled that failed to complete the 5-visit study protocol.

^4^ Cats where no practice or country information was recorded.

At each practice, posters were displayed prominently in waiting room areas to alert cat owners to the study. Owners who expressed an interest were eligible if their cat met the eligibility criteria of the study (below), they could easily be contacted by telephone or email and consented to presenting their cat for five weight loss assessments. Cats were eligible for enrolment if they were skeletally mature (i.e. at least 12 months of age), overweight (e.g. body condition score [BCS] of ≥ 7/9, see below), and were otherwise in good health. Female cats could not be pregnant or lactating at the time of enrolment. Further, all cats had to be indoor, not living with any other cats, and used to consuming either a commercial dry or wet food exclusively or a mix of wet and dry food. In addition, cats were not allowed to have any prior history of adverse reaction to food or be on a therapeutic diet (other than a weight loss diet), could not be suffering from a concurrent disease (e.g. hyperthyroidism, systemic disease such as diabetes mellitus, chronic kidney disease etc). Finally, although prophylactic treatments, including vaccination, ecto- and endo-parasiticides were allowed, no therapy that might feasibly influence the weight loss process were permitted (e.g. glucocorticoid therapy, anticonvulsants, appetite stimulants, antibacterials, insulin).

After enrolment, if cats were subsequently excluded, the reasons for suspension were recorded, for example failure to return for appointments, failing to consume the diet due to food refusal or food aversion, and not complying with the study protocol. Cats could also be withdrawn if they developed an unrelated illness during the study, with the decision being made after assessment by the attending veterinarian. In such circumstances, the veterinarian considered the nature and severity of the illness, the treatment that might be required, welfare of the patient, and the wishes of the owner. When a cat with a concurrent illness was permitted to continue, short-term treatments were allowed such as non-steroidal anti-inflammatory drugs and antibacterials. The use of such treatments was also recorded in the study notes.

### Weight loss foods

Three foods were used in the study (Table 2) all of which were high in protein and fibre and were formulated to meet essential nutrient requirements when fed for weight loss (dry food: Satiety Weight Management; wet food: Obesity Management, Royal Canin, Aimargues, France; Table 2). Cats could either be fed dry food exclusively, wet food exclusively, or a mix of wet and dry food, and the choice was dependent upon the preferences of both owner and cat.

**Table 2:** Average composition of diets for weight loss.

*Criterion*	Dry food ^a^		Wet food ^b^			
			Obesity Weight Management		Calorie control	
*ME content*	3140 kcal/kg		640 kcal/kg		650 kcal/kg	
	Per 100g AF	g/1000 kcal (ME)	Per 100g AF	g/1000 kcal (ME)	Per 100 g AF	g/1000 kcal (ME)
*Moisture*	5.5	18	84.0	1300	84.0	1294
*Crude protein*	34.0	108	7.5	116	8.0	123
*Crude fat*	9.0	29	2.0	31	1.8	28
*Nitrogen-free extract* ^c^	28.8	92	3.1	48	4.1	62
*Digestible carbohydrate* ^d^	19.1	61	3.1	48	3.2	49
*Crude fibre*	13.9	44	1.5	23	1.0	15
*Total dietary fibre*	23.6	75	1.5	23	1.8	28
*Ash*	8.8	28	1.9	29	1.2	19
*Calcium*	1.3	4.2	0.3	4.2	0.2	2.3
*Phosphorus*	1.2	3.7	0.2	3.4	0.2	2.3
*Sodium*	0.6	1.9	0.2	2.5	0.1	1.7
*Potassium*	1.1	3.6	0.2	2.5	0.2	2.8
*Chloride*	1.0	3.2	0.2	3.1	0.2	2.8
*Fibre sources*	Cellulose, chicory pulp, psyllium husk, diet cereals		Cellulose, carrageenan, guar		Cellulose, carrageenan, guar	

AF: As fed; ME: metabolisable energy calculated using a predictive equation based on total dietary fibre.

^a^ High protein high fibre dry food (Satiety Weight Management, Royal Canin);

^b^ High protein high fibre wet food (Calorie Control [North and South America] or Obesity Weight Management [Europe, Asia, Africa], Royal Canin).

^c^ Nitrogen-free extract (NFE) calculated using the following predictive equation: NFE [g] = Dry matter [g]—(crude fat [g] + crude protein [g] + ash [g] + crude fibre [g]).

^d^ Digestible carbohydrate fraction (e.g. sugars and starch) calculated in a similar manner to NFE but using total dietary fibre rather than crude fibre: digestible carbohydrate [g] = dry matter [g]—(crude fat [g] + crude protein [g] + ash [g] + total dietary fibre [g]). Therefore, this is a more accurate measure of the soluble carbohydrate fraction than NFE.

### Measurements

A series of repeated measurements were made in all cats during the study, and these included bodyweight, body condition score (BCS), activity, quality of life (QOL), and food-seeking behaviour. To ensure consistency the same study investigator performed all measurements from a single cat throughout the study. Bodyweight was measured using electronic weigh scales, with the exact make and model varying by practice. A 9-integer unit body condition score (BCS) was used to determine body condition [23]. To improve consistency with body condition scoring, Royal Canin representatives provided BCS training for any study investigator that required it; decisions about the provision of BCS training were made on an individual basis after a discussion with the practice. Study investigators also assessed activity and QOL after discussions with the owner using a subjective scoring system (Table 3). In deciding on the most appropriate score (e.g. -1, 0, 1), at least two characteristics from a score were required for it to be selected. The investigator also assessed food-seeking behaviour using a similar subjective scoring system (Table 3). Here, different types of food-seeking behaviour were first discussed with the owner to determine whether it was present. When such behaviours were reported, their relationship to feeding was then determined.

**Table 3:** Criteria for subjective determination of activity, quality of life and food seeking behaviour in study cats.

Assessment	Scoring	Description
Activity	-1	My cat is not active
		My cat spends most of the time sleeping
		My cat is not playful
		My cat seems to have difficulty climbing, jumping up and off high places (kitchen counter, cat tree…)
	0	My cat is somewhat playful
		My cat can climb to some high places
	+1	My cat is always ready to play
		My cat is able to climb, jump on and off any high places (kitchen counter, cat tree,…)
Quality of life	-1	My cat seems anxious
		My cat is not actively exploring her/his environment and is not playful
		My cat exhibits increased hiding or often attempts to hide
		My cat spends a greater proportion of her/his time awake (e.g. exhibiting vigilance and scanning behaviour)
		My cat shows signs of irritability towards some people or pets
		My cats finds it difficult to groom its back
		My cat finds it difficult to climb, jump up and off high places (kitchen counter, cat tree…)
		My cat is reluctant to be stroked and handled
	0	My cat does not seem anxious
		My cat is shy and tends to hide sometimes
		My cat is slightly irritable towards some pets or people
		My cat is able to climb to some high places
	+1	My cat seems to enjoy life
		My cat does not show any signs included in score “-1”
Food seeking behaviour		Descriptions of food-seeking behaviours: • My cat acts as if they are hungry • My cat consumes food very rapidly and 'greedily' • My cat vocalises (e.g. meows) for more food • My cat attempts to steal food, to steal from the dustbin, and/or to open doors of cupboards containing food • My cat wakes me up at night asking to be fed • My cat follows me everywhere and appears 'clingy' • My cat very often turns over their bowl after meals or between meals • My cat is irritable and sometime aggressive
	0	One or more food-seeking behaviours observed just before meals
	- 1	One or more food-seeking behaviours observed just before meals, and occasionally between meals
	- 2	One or more food-seeking behaviours observed just before meals, often between meals, and right after meals
	- 3	One or more food-seeking behaviours observed just before meals, constantly between meals, and right after meals

At each visit, study investigators recorded scores for activity, QOL, and food-seeking behaviour after discussions with the owner using a subjective scoring system.

### Weight loss protocol

At each participating practice, cases were enrolled during an initial 4-month period, and this was followed by a period of approximately 3 months to allow all participating cats to complete their weight loss (Table 4). Each cat visited the practice on 5 occasions, comprising an initial assessment and enrolment visit (visit 1) and 4 follow-up visits (visits 2 to 5).

**Table 4:** Details of the 3-month study protocol.

Visit	1	2	3	4	5
Time (weeks)^1^	0	2	4	8	12
Body weight	X	X	X	X	X
Body Condition Score	X				
Target Body Weight	X				
Initial Energy Allocation	X				
Allocation Adjustment		X	X	X	
Activity	X	X	X	X	X
Quality of Life	X	X	X	X	X
Food-seeking behaviour	X	X	X	X	X

^1^ Recommended time of visits in weeks as per the study protocol.

Study investigators recorded scores for activity, QOL, and food-seeking behaviour after discussions with the owner using a subjective scoring system (see Table 3).

At visit 1, a history was taken and physical examination performed to confirm the eligibility of the cat for the study (see above). The cat was then weighed and their BCS assessed. The attending veterinary professional then subjectively assessed activity, QOL, and food-seeking behaviour after a discussion with the owner (Table 3). Target body weight (TBW) and initial daily food energy allocation were then determined using similar methods to those previously described [20,23–25]. Briefly, TBW was determined for cats with a BCS of 7, 8, and 9, the current bodyweight was divided by 1.2, 1.3 and 1.4, respectively. Initial daily food energy allocation was calculated as 222 kJ per kg^0.711^ of TBW (53 kcal per kg^0.711^ of TBW) per day, a formula adapted from energy intakes known to induce controlled weight loss in previous feline weight loss studies using the same or similar diets [19,20]. For consistency and simplicity, an internet-based computer programme specifically developed for the trial (VET FOLLOW UP, Royal Canin) was utilised by study investigators. Details of sex, neuter status, current body weight, and BCS were first entered and the programme then automatically calculated TBW and starting food energy allocation. The programme also automatically converted the starting food energy allocation into a daily ration (in grams or tins of food), based on the food type selected (e.g. dry or mix of wet and dry food). For dry food, in all countries apart from the USA, portion size was accurately determined using electronic gram scales wherever possible. If owners refused to use electronic gram scales, a calibrated measuring cup was instead used: here, the cup was individually calibrated by first weighing a portion of food on gram scales at the practice, and marking portion size on the cup using an indelible marker pen. For the USA, owners were provided with an 8 US fl oz cup (237 mL) with marked graduations corresponding to one quarter, one third, one half, three-quarters and one cup. All food portions were provided in ounces, and a conversion provided for the corresponding number of cups. The owners were instructed to divide the daily ration into at least two daily meals, with food given in the morning and evening.

The optimal timing of the four follow-up visits was 2 weeks (visit 2), 4 weeks (visit 3), 8 weeks (visit 4), and 12 weeks (visit 5), and study investigators attempted to ensure that cats were seen at these times, although flexibility was allowed with timings depending on owner availability. Measurements were taken at each follow-up visit according to the study protocol including bodyweight activity, QOL and food-seeking behaviour (Table 4). However, whilst veterinary professionals could consider changes in BCS to help monitor progress during the study, this measure was not included as an outcome, based on an extrapolation of the results of a recent canine study that highlighted its insensitivity for detecting changes in body composition [25]. In this study, a change of 10–15% body weight is typically required for a 1-unit change in BCS) [25]. Compliance with the weight loss programme was also discussed at each visit, for example whether they had measured food accurately and whether the cat had obtained additional food (e.g. treats or table scraps fed by the owner or food stolen by the cat).

The internet-based computer software used to determine TBW and initial food allocation was also used to monitor weight loss; study investigators entered body weights at each visit, and the software automatically calculated the rate of weight loss. The software also suggested adjustments to the feeding plan to aim for a weekly weight loss of between 0.5% and 2% per week. For example, if the rate of weight loss had been <0.5% per week, a 10% reduction in energy allocation was suggested, whilst a 10% increase in energy allocation was recommended if weight loss had been faster than 2% per week. However, the study investigator could use their judgement on adjusting the ration, based upon the individual circumstances of the case. Examples of reasons for overriding the recommended adjustment included evidence of non-compliance (which could be corrected by owner counselling) and an owner being reluctant to change the food allocation.

### Data handling and statistical analysis

#### Sample size

Sample size was not calculated formally and, instead, practices were simply asked to recruit as many cats as possible to ensure that the study was as large as feasible for the time frame.

#### Data handling and computer software

The internet-based software (VET FOLLOW UP, Royal Canin) used by study investigators for TBW, food allocations and weight monitoring was also used for data recording. A dataset containing all study data are available as supporting information (S1 Dataset). Data were analysed with 2 computer software packages (SAS version 9.3, SAS Institute Inc., USA; Stats Direct version 3.0.171, Stats Direct Ltd., UK). Continuous data are reported as mean ±SD, when normally distributed, and as median and interquartile range (IQR) or median and range when not normally distributed, and the level of statistical significance was set at *P*<0.05, for 2-sided analyses.

#### Missing data

The restricted maximum likelihood (RML) method was applied to linear mixed models to deal with missing data. Age data were missing for 5 cats, energy allocation data were not reported for a total of 67 instances in 21 cats, whilst body weight measurements of 11 cats were missing (7 at Visit 2, 3 at Visit 3 and 2 at Visit 4). Data from behavioural questionnaires were complete.

#### Rate of weight loss

Changes in rate of weight loss were assessed using linear mixed models with visit number as a fixed effect and cat as a random term. If residuals were not normally distributed, quantitative variables were rank transformed. Given that several visits were considered, Scheffé’s method was used to adjust the results of post-hoc analyses to avoid alpha-risk inflation.

#### Factors associated with study completion

Simple and multiple logistic regression were used to determine the association between study completion and a range of explanatory variables. The outcome variable was whether the cat completed all 5 visits after enrolment or did not complete the trial, whilst the explanatory variables were age at first visit, breed (pedigree vs. mixed breed), sex, neuter status, start bodyweight, target body weight, initial BCS, average energy intake during the study, food type (dry food only versus either wet or mixed feeding), and geographical region. For geographical region, 4 dummy variables were created to enable 5 different regions to be tested (North America [reference category], Asia, Europe, and South America); Africa was not included in the analysis given the small number of cats enrolled. Initially, all variables listed above were tested separately with simple logistic regression. A multiple logistic model was then built, which initially included the variables identified as *P*<0.2 in simple regression. The model was then refined using backwards-stepwise elimination, of the least significant variable, and variables were only retained in the final model if they were significant in their own right (*P*<0.05). A bootstrap procedure, involving 1000 random resamplings, was used to cross-validate the confidence intervals calculated for odds ratios (OR) derived from the fitted logistic models [26,27], and the results reported are bias-corrected OR along with 95% confidence intervals (95% CI) and with the associated *P* value.

To explore the association between completion and geographical location further, the proportions of cats completing in 4 different regions (Asia, Europe, North America, South America) and also in the countries which had at least 20 recruiting practices and at least 50 cats (Argentina, Germany, Italy, Portugal, UK and USA) were compared using Fisher’s exact tests. Initially, the proportions completing in all geographical regions and all countries were compared simultaneously using the ‘r by c Fisher’ function in Stats Direct. When the r by c Fisher function returned a significant result, post-hoc comparisons were made by comparing different geographical regions and different countries using 2 × 2 Fisher’s exact tests. A Bonferroni correction was applied to these 2 × 2 comparisons in order to reduce the risk of alpha inflation. Since 6 post-hoc 2 × 2 Fisher’s exact tests were required to compared different geographical regions, all *P* values were multiplied by 6 before interpretation; similarly, for country, all *P* values were multiplied by 15 before interpretation. Bonferroni-corrected *P* values are reported in the results for the post-hoc Fisher’s exact tests.

#### Factors associated with percentage of weight lost

Stepwise linear regression analyses were used to assess the impact of various fixed effects (age, breed, sex, neuter status, initial BCS, average energy intake during the study, and geographical location [within Europe vs. outside Europe]) on percentage body weight loss in the 413 cats that had completed the study (S2 Dataset). Normality of residual distributions were checked, and data were rank transformed if appropriate. Contingency tables were used to assess the independence of the effect of each factor on percentage weight loss compared with all other factors. Stepwise regressions were set in such a way that each forward selection step could be followed by a backward elimination step if necessary using the Schwarz Bayesian Information Criterion (SBC) [28,29]), as previously described [30]. Complementary analysis of baseline data was performed using a classic general linear model (GLM) to assess the effect of the most relevant factors identified in the stepwise regression. For the GLM analysis, residual distributions were checked for normality and were subsequently rank transformed if not normal. To explore the findings from GLM further, a Kruskal-Wallis test was used to compare percentage weight loss both amongst 4 different geographical locations (Asia, Europe, North America, South America) and also amongst countries which had at least 20 recruiting practices and at least 60 cats (Argentina, Germany, Italy, Portugal, USA). Post-hoc comparisons were made using the Dwass-Steel-Chritchlow-Fligner test.

#### Changes in the behavioural observations of the owner

Statistical analyses of subjective scores of activity, QOL and food-seeking behaviour (rank transformed to be treated as ordinal data) were conducted using linear mixed models with visit number, age (coded in quartiles with quartile 1 representing the youngest cats, and quartile 4 representing the oldest cats), initial body condition (overweight [BCS 7] vs obese [BCS 8 and 9]) and their related interaction as fixed effects, and cat as a random term. Given that several visits were considered, Scheffé’s method was again used to adjust post-hoc analyses to avoid the risk of alpha inflation.

## Results

### Details of participating countries, practices, and cats

**Fig 1:**
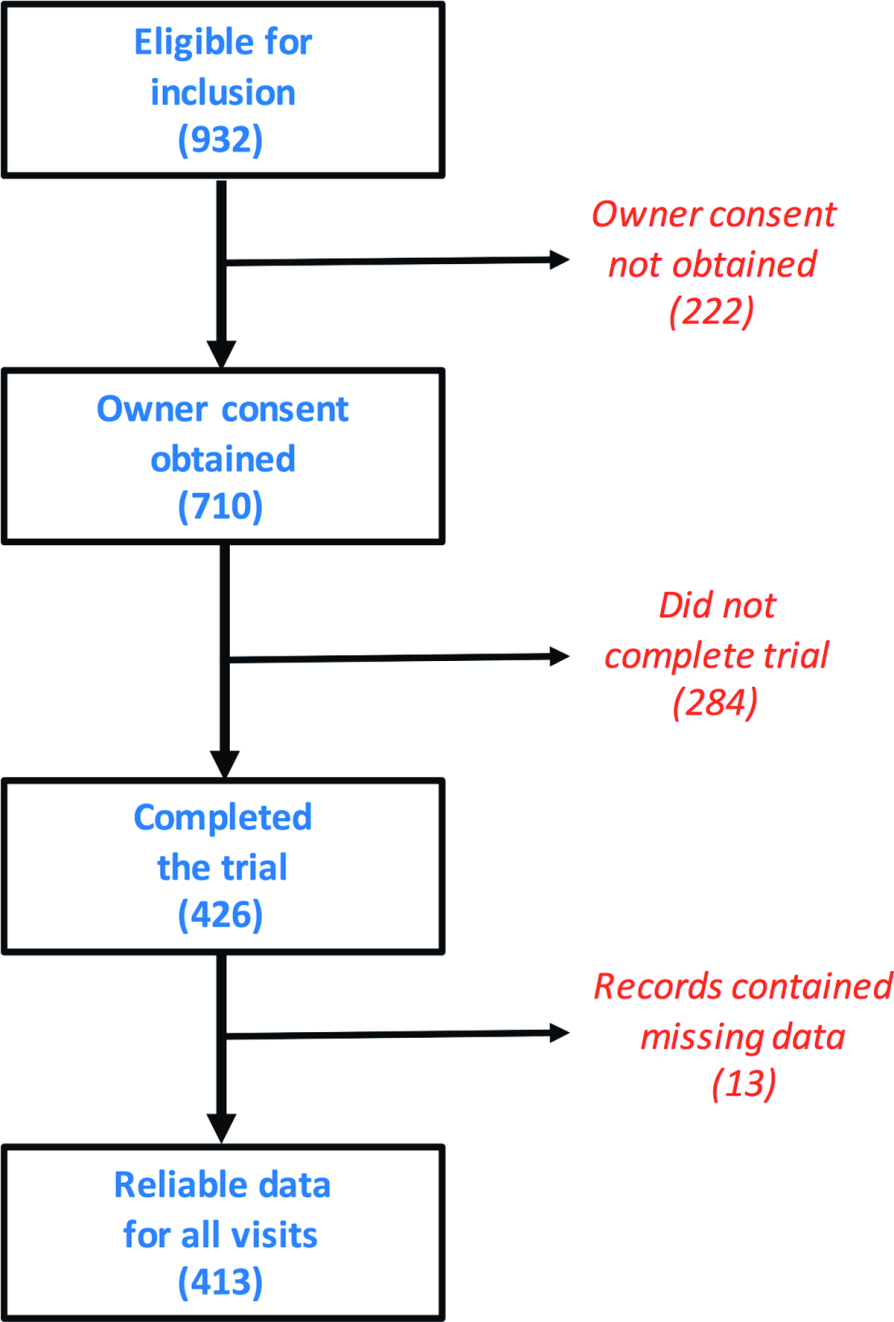
Flow diagram depicting the outcomes of the cats eligible for the study.

A total of 306 veterinary practices from 25 countries participated (Table 1) across 5 different geographical locations, with a median of 4 practices per country (IQR 3–12). After initial screening, 932 cats were eligible, and 710 of owners consented to their cat participating (Fig 1). Of those that started the weight loss trial, 426 cats (60%) completed the full 3 months of weight loss attending all visits, and complete data for all 5 visits were available for 413 cats (58%) (Table 1). Of the 284 cats that did not complete the trial, 244 cats (86%) failed to return for revisits and were lost to follow up, 11 (4%) stopped due to owner non-compliance, 1 (<1%) stopped because of a concurrent medical condition, 4 (1%) stopped because the cat refused to eat the food, and 3 (1%) owners found the programme too tough to follow. The reasons why the remaining 21 cats (7%) stopped were not recorded.

Of the final population of 413 cats (Table 5), 186 (45%) were female (175 neutered) and 227 cats (55%) were male (220 neutered). The cats comprised 14 different breeds, with 311 cats (75%) being of mixed or unknown breed. The most common pedigree breeds represented were European shorthair (42, 10%), American shorthair (27, 6%) and British shorthair (15, 4%). Age data were available for 388 cats and, within this group, the median age was 72 months (range 12 to 200 months). Median starting body weight of the cats was 6.7 kg (range 2.8 to 15.0 kg) and median BCS was 8 (range 7 to 9).

**Table 5:** Details of the cats enrolled in and completing the 12-week weight loss trial.

Variable		Enrolled ^1^		Completed ^2^
**Number of cats**		710		413
**Sex**				
Intact male		22 (3%)		7 (2%)
Neutered male		372 (52%)		220 (53%)
Intact female		13 (2%)		11 (3%)
Neutered female		303 (43%)		175 (42%)
**Median age (IQR) (months)**		73 (12 to 179)		72 (12 to 179)
**Breed**				
Mixed/unknown breed			510 (72%)	311 (75%)
European shorthair			83 (12%)	42 (10%)
American shorthair			35 (5%)	27 (7%)
British shorthair			27 (4%)	15 (4%)
Other breeds			55 (8%)	18 (6%)
**Median starting weight (IQR) (kg)**		6.6 (5.7 to 7.7)	6.7 (5.8 to 7.6)
**Body condition score** ^3^			
7/9		203 (28%)	122 (30%)
8/9		304 (43%)	166 (40%)
9/9		203 (29%)	125(30%)
**Weight loss diet**			
Dry food only		515 (73%)	309 (75%)
Mix of dry and wet food		187 (26%)	105 (25%)
Wet food only		4 (<1/%)	2 (<1%)

^1^ Cats that were eligible and whose owners consented to them being enrolled in the trial.

^2^ Cats enrolled that completed the whole 5-visit study protocol.

^3^ Body condition score determined using a 9-integer unit system [19]. IQR: inter-quartile range.

## Weight loss food and dietary energy allocation

Of the 413 study cats, 309 (75%) were initially fed dry food exclusively (median weight 6.7 kg, range 2.8–15.0 kg), 102 (25%) were fed a mix of wet and dry food (median weight 6.7 kg, range 3.8–11.2 kg), and the remaining 2 (<1%) were fed wet food exclusively (median weight 5.9 kg, range 5.7–6.1 kg). The mean ± SD starting allocation for the study cats was 222 ±6.7 kJ/kg^0.711^/day TBW (53 ±1.6 kcal/kg^0.711^/day TBW) and 211 ±36.3 kJ/kg^0.711^/day TBW (50 ±8.6 kcal/kg^0.711^/day TBW) at the end of study. The mean energy allocation for the whole of the 12 weeks was 219 ±20.7 kJ/kg^0.711^/day TBW (52 ±4.9 kcal/kg^0.711^/day TBW).

### Weight loss outcomes in successful cats

Of the 413 cats that completed the study, 402 (97%) lost weight, 6 (1%) maintained a stable weight (i.e. within ±1% of starting body weight), and 5 (1%) gained >1% weight. A total of 402 (97%), 339 (82%), 204 (49%), and 36 (9%) of cats lost more than 1%, 5%, 10% and 20% of their starting bodyweight, respectively. By the end of the study 22/413 cats (5%) had reached target body weight and were in ideal body condition, as judged by BCS performed by the attending veterinary professional. Although the intention for follow up visits was to have 4 over 12 weeks, there was considerable deviation from this, with median (IQR) time from starting the programme (visit 1) being 15 days (14–19 days), 33 days (28–43 days), 61 days (56–72 days), and 91 days (84–103 days) for visits 2, 3, 4 and 5, respectively.

The mean (±SD) bodyweight lost was 3.0±3.0% (by visit 2), 5.5±4.2% (by visit 3), 8.1 ±5.2% (by visit 4) and 10.6% ±6.3% (by visit 5). The mean (±SD) rate of weight loss for the whole of the study was 0.8 ±0.50% of initial bodyweight per week, although the rate decreased sequentially over visits 2 to 4 (visit 1 to 2: 1.2 ±1.44%/week; visit 2 to 3: 1.0 ±1.77%/week; visit 3 to 4: 0.7 ±1.19%/week; *P*<0.001), but remained similar thereafter (visit 4 to 5: 0.7 ±1.28%/week).

### Factors affecting the outcome of weight loss

Factors associated with the odds of cats completing all study visits were explored by simple and multiple logistic regression. On simple regression, three factors were identified: pedigree status, diet type, and geographical region (Table 6), and these were added to the initial multiple regression model. After the initial model was refined by backward-stepwise elimination, diet type and geographical region remained in the best-fit model (Table 7). For diet type, being fed either wet food or a combination of wet and dry was negatively associated with the odds of completing the study, although the effect size was relatively small with relatively wide confidence intervals (OR 0.69, 95%CI 0.48–0.95, *P* = 0.036). For geographical location, cats from North America were more likely to complete (OR 2.20, 95%CI: 1.38–3.61, *P*<0.001) and cats from Asia were less likely to complete (OR 0.29, 95%CI: 0.12–0.60, *P* = 0.002) than cats in Europe. Using Fisher’s exact tests, a difference amongst geographical region was noted (*P*<0.001); post-hoc analysis revealed that a greater proportion of cats completed the study in North America compared with Asia (*P*<0.001) and Europe (*P* = 0.012), and a greater proportion of cats completed in Europe compared with Asia (*P* = 0.006); no other differences in the proportion of cats completing amongst geographical regions. Differences in the proportion of cats completing the study were also noted amongst countries (*P*<0.001); post-hoc analysis revealed that a greater proportion of cats completed in Germany than in Argentina (*P*<0.001), Italy (*P*<0.001), Portugal (*P*<0.001), UK (*P*<0.001) and USA (*P* = 0.008); a greater proportion of cats completed in USA compared with Argentina (*P* = 0.011), Italy (*P*<0.001), and the UK (*P*<0.001); and a greater proportion of cats completed in Italy compared with the UK (*P*<0.001).

**Table 6:** Simple logistic regression analysis of factors associated with completing the trial.

Logistic regression	Odds of completing the trial		
	OR ^1^	95% CI ^2^	P value
**Age at first visit (months)**	1.000	0.998–1.003	0.612
**Sex**			
Female		Reference category ^3^	
Male	0.950	0.704–1.283	0.738
**Neuter Status**			
Entire		Reference category ^3^	
Neutered	0.750	0.380–1.482	0.408
**Breed**			
Not pedigree		Reference category ^3^	
**Pedigree**	**1.561**	**1.072–2.274**	**0.020**
**Initial bodyweight (kg)**	1.000	0.923–1.083	0.996
**Target bodyweight (kg)**	0.962	0.860–1.075	0.492
**Body condition score**			
7/9		Reference category ^3^	
8/9	0.799	0.557–1.145	0.221
9/9	1.064	0.714–1.585	0.760
**Dietary energy allocation (kcal/day)**	0.999	0.996–1.004	0.995
**Food type**			
Dry food		Reference category ^3^	
**Wet food or mixed feeding**	**0.802**	**0.574–1.121**	**0.197**
**Geographical region**			
Europe		Reference category ^3^	
**Asia**	**0.312**	**0.146–0.667**	**0.003**
**North America**	**2.129**	**1.362–3.328**	**<0.001**
South America	0.820	0.492–1.368	0.448

^1^ OR: odds ratios;

^2^ 95% CI: ninety-five percent confidence interval.

^3^ Reference category used in logistic regression.

Variables highlighted in bold qualified for inclusion in the multiple regression analysis at *P* <0.20 (Table 7).

**Table 7:** Multiple logistic regression analysis of factors associated with completing the trial.

Logistic regression	Odds of completing the trial		
	OR^1^	95% CI^2^	P value
**Food type**			
Dry food		Reference category ^3^	
Wet food or mixed feeding	0.686	0.479–0.948	0.036
**Geographical region**			
Europe		Reference category^3^	
Asia	0.291	0.125–0.598	0.002
North America	2.199	1.383–3.608	<0.001
South America	0.734	0.429–1.228	0.246

^1^ OR: odds ratios;

^2^ 95% CI: ninety-five percent confidence interval.

^3^ Reference category used in logistic regression.

Variables highlighted in bold qualified for inclusion in the multiple regression analysis at *P* <0.20 (Table 6).

**Fig 2:**
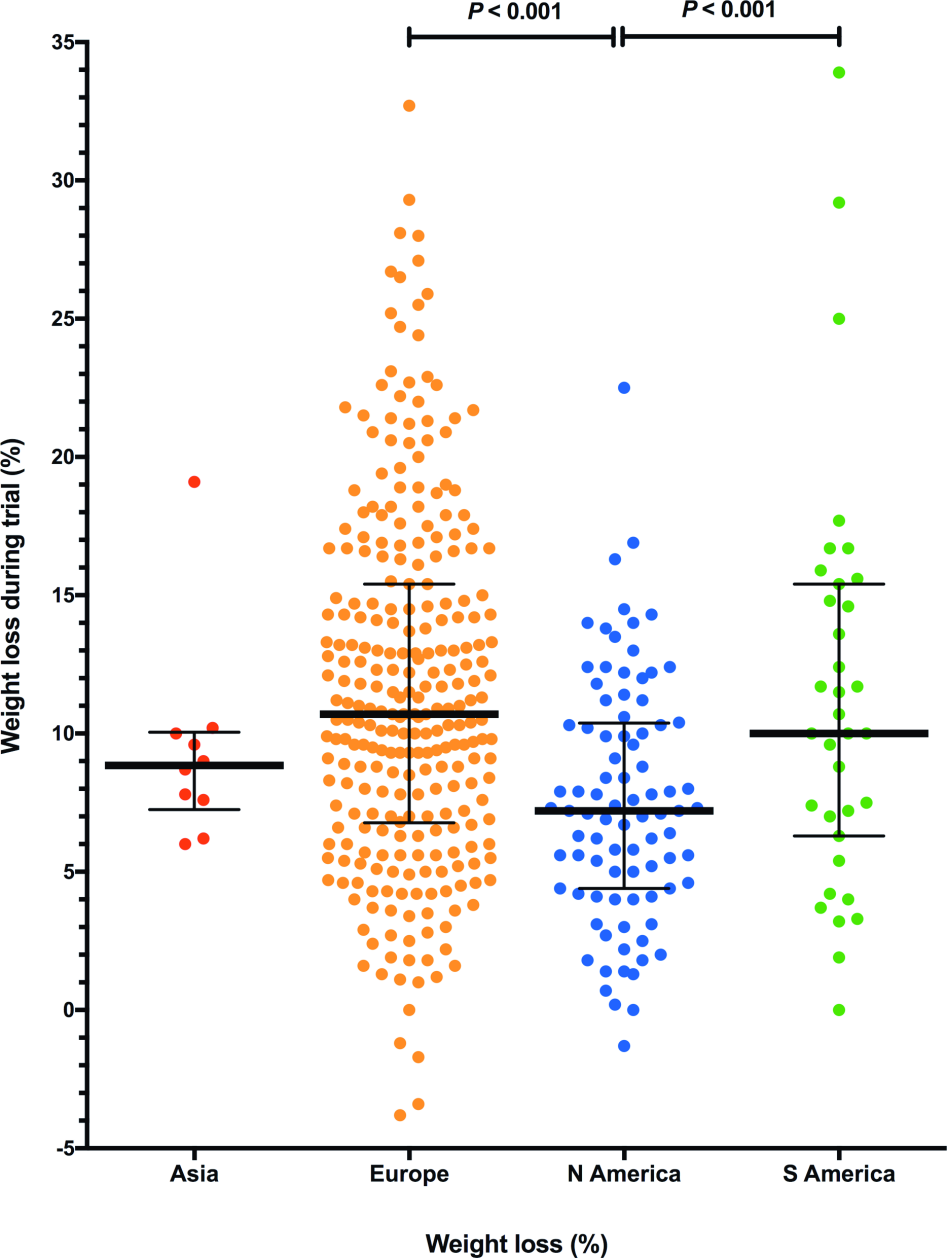
Dot plots comparing percentage weight change in cats from different geographical locations over the 3-month study. Individual cats are shown by the coloured circles, the thick horizontal lines represent the median for each geographical location, and the error bars represent the inter-quartile range (top and bottom of box).

Factors associated with the percentage weight lost were then explored using stepwise linear regression. Only geographical location was retained in the final model, whereby percentage weight loss within Europe (median 11.5% weight loss, IQR 6.9–15.3%) was greater than in the rest of the world (median 8.7% weight loss, IQR 5.2–11.5%, *P*<0.001; Fig 2). No other factors improved the quality of the final model. The influence of geographical location was explored further by comparing median percentage weight loss amongst different continents and countries. There was a highly significant difference in median percentage weight loss both amongst different geographical regions (*P*<0.001) and countries (*P*<0.001). For geographical region, post-hoc analysis demonstrated that median percentage weight loss was greater in Europe and South America compared with North America (Fig 2); for country, post-hoc analysis revealed that median percentage weight loss was greater in Argentina, Germany and Italy compared with the USA, whilst median percentage weight loss was greater in Germany and Italy compared with Portugal. There were no other significant differences in percentage weight loss amongst geographical regions or individual countries.

### Activity

**Fig 3:**
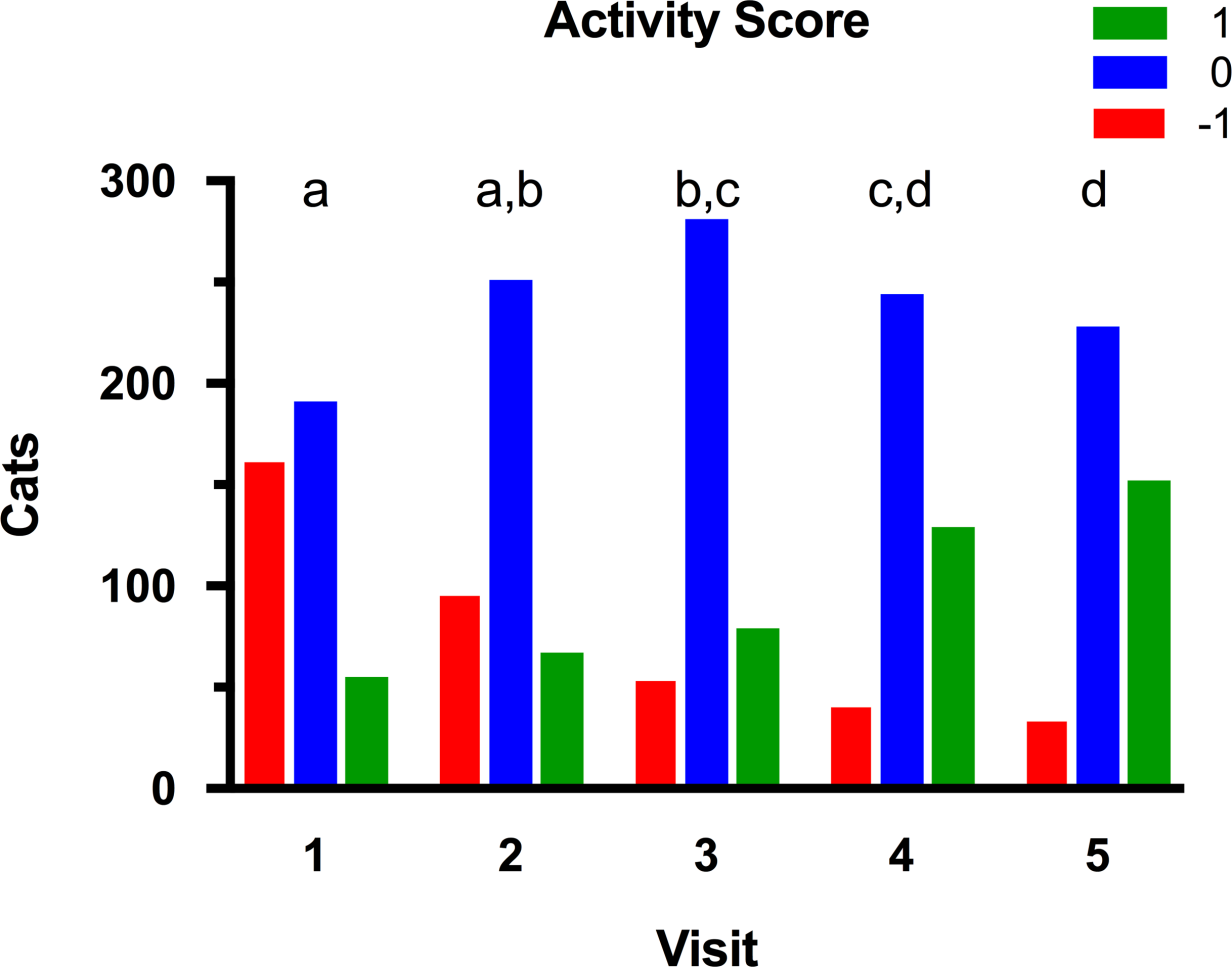
Activity scores for cats during the study. Activity score was subjectively determined at each visit after a discussion between the veterinarian and owner (Table 3). At each visit, blocks with different colours represent the proportion of cats assigned an activity score of -1 (red), 0 (blue) and 1 (green), respectively. Visits with different superscripts (a, b, c and d) are significantly different from one another at *P*<0.05.

At the initial visit, 167 (40%), 191 (46%), and 55 (13%) cats had an owner-reported activity score of -1, 0 and 1, respectively (Fig 3). Overweight cats (BCS 7/9) had significantly greater activity scores than cats that were obese (8-9/9) at baseline (*P* = 0.002), whilst owner-reported activity decreased with age (*P*<0.001). During the study, owner-reported activity improved in 202 cats (49%), stayed the same in 188 cats (46%), and worsened in 23 cats (6%). Using a linear mixed models analysis, both age (*P*<0.001) and time (i.e. visit number, *P*<0.001; Fig 3) had independent effects on owner-reported activity, and there was also a significant time-age interaction (*P* = 0.002). For time, post-hoc analysis revealed significantly improved activity for visits 3, 4 and 5, compared with visit 1. Activity was also improved at visits 4 and 5 compared with visit 2, and for visit 5 compared with visit 3. For age, cats in quartiles 1 and 2 (youngest) were more active than cats in quartile 4 (oldest), whilst cats in quartile 1 were also more active than cats in quartile 3. On further analysis of the time-age interaction, and in contrast to all other visits, cats in age quartile 2 were more active than cats in quartile 1 at visit 5 only.

### Quality of life

**Fig 4:**
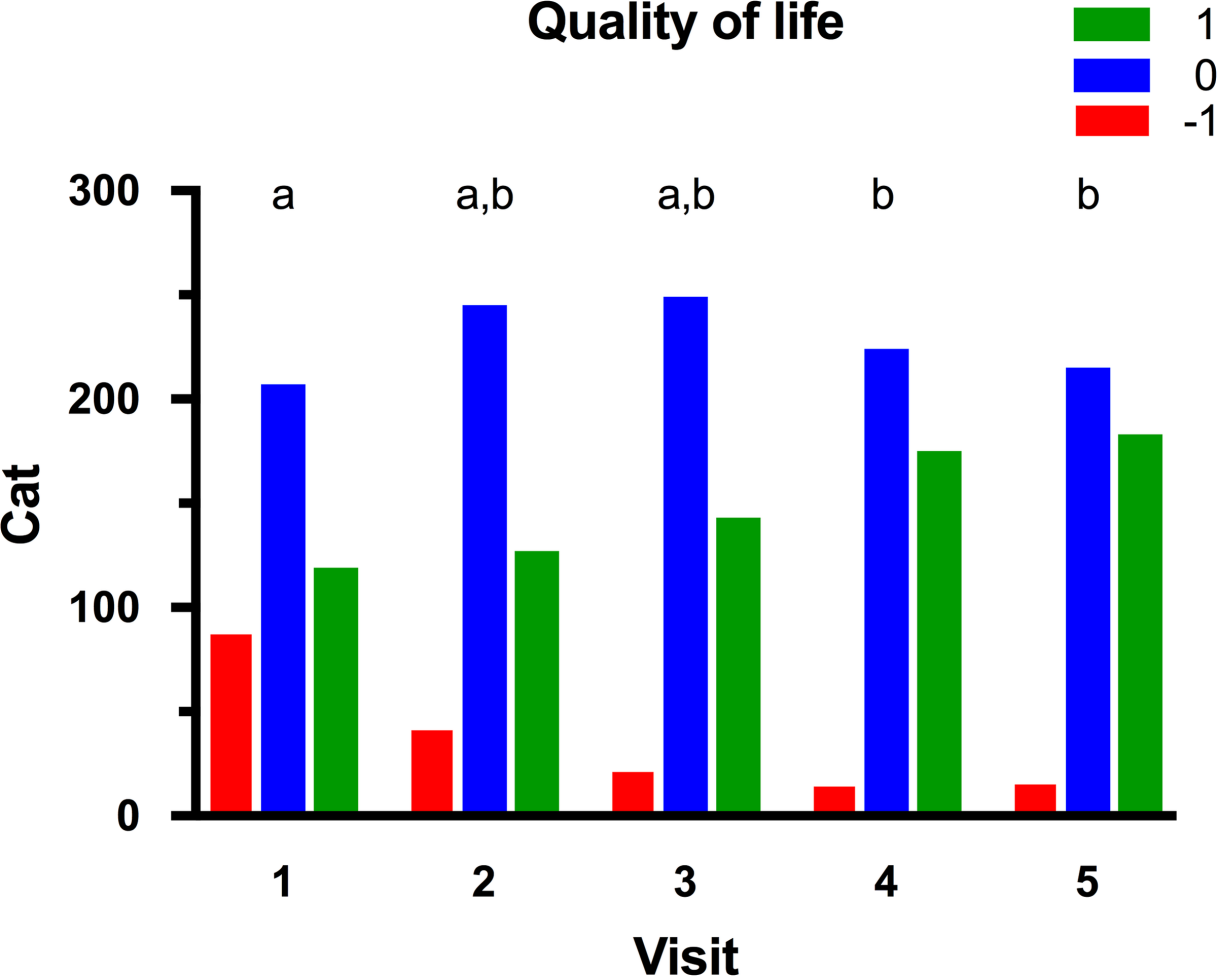
Quality of life (QOL) scores for cats during the study. QOL was subjectively determined at each visit after a discussion between the veterinarian and owner (Table 3). At each visit, blocks with different colours represent the. proportion of cats assigned an activity score of -1 (red), 0 (blue) and 1 (green), respectively. Visits with different superscripts (a, b, c and d) are significantly different from one another at *P*<0.05.

At the initial visit, 87 (21%), 207 (50%), and 119 (29%) cats had an owner-reported QOL score of -1, 0 and 1, respectively (Fig 4). There was no difference in QOL scores between overweight and obese cats at baseline (*P* = 0.301), but QOL was worst for the oldest cats (quartile 4; *P* = 0.013). During the study, owner-reported QOL improved in 158 cats (38%), stayed the same in 204 cats (49%), and worsened in 51 cats (12%). Using a linear mixed models analysis, both age (*P*<0.001) and time (i.e. visit number, *P*<0.001; Fig 3) had independent effects on owner-reported QOL but, unlike for activity scores, there was no interaction between them. For time, post-hoc analysis revealed significantly improved QOL for visits 4 and 5 compared with baseline (Fig 4), whilst QOL was significantly worse for the oldest cats (quartile 4) than for younger cats (quartiles 1–3).

### Food-seeking behaviour

**Fig 5:**
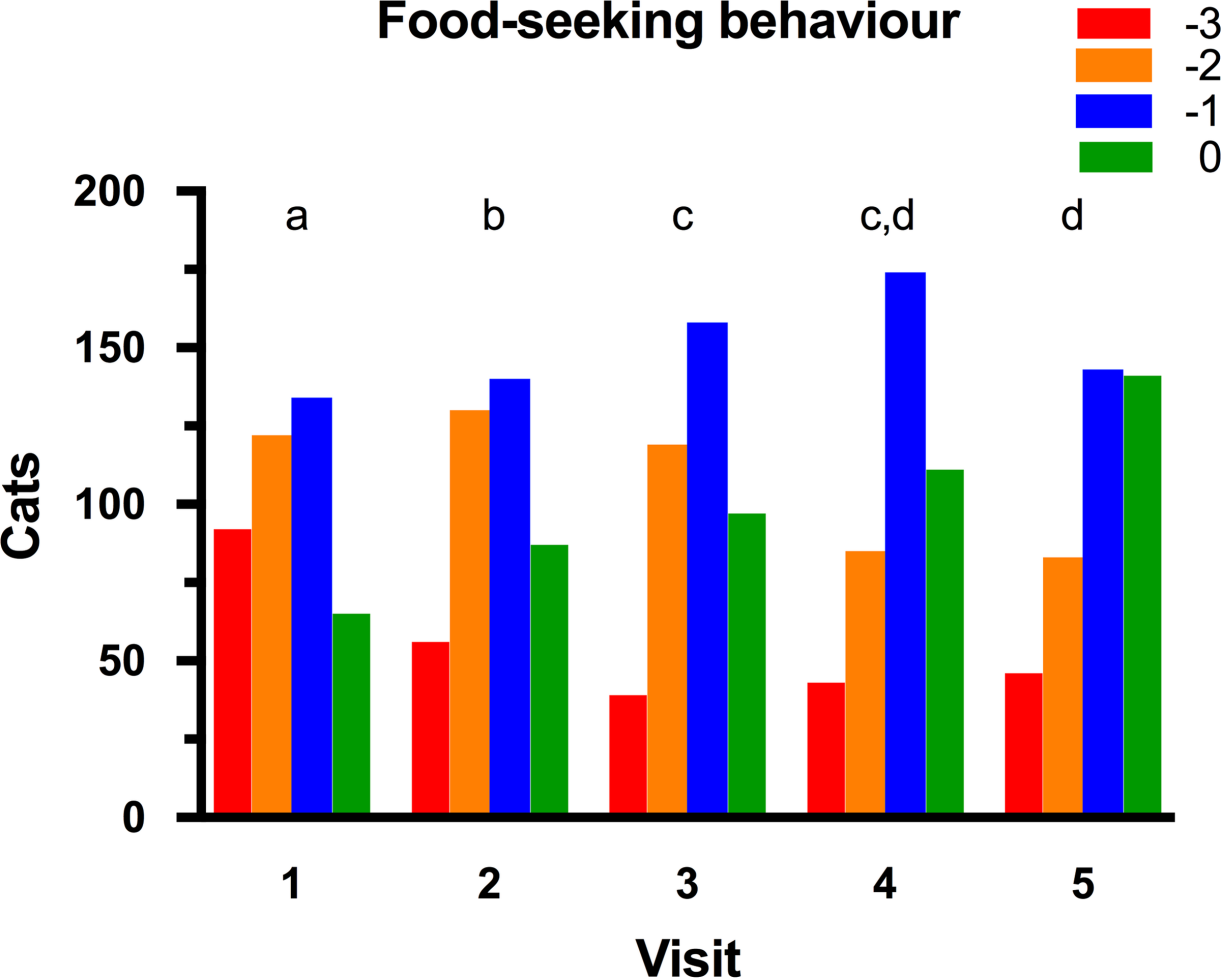
Food-seeking behaviour scores for cats during the study. Food seeking behaviour was subjectively determined at each visit after a discussion between the veterinarian and owner (Table 3). At each visit, blocks with different colours represent the proportion of cats assigned an activity score of 0 (red), -1 (orange), -2 (blue) and -3 (green), respectively. Visits with different superscripts (a, b, c and d) are significantly different from one another at *P*<0.05.

At the initial visit, 92 (22.3%), 122 (29.5%), 134 (32.4%), and 65 (15.8%) cats had an owner-reported food-seeking behaviour score of -3, -2, -1, and 0, respectively (Fig 5). Further, there was no effect of either age (*P* = 0.799) or body condition (overweight vs. obese, *P* = 0.478) on food-seeking behaviour. During the study, owner-reported food-seeking behaviour decreased in 200 cats (48%), stayed the same in 140 cats (34%), and worsened in 73 cats (18%). A linear mixed models analysis revealed a significant decrease in food-seeking behaviour overall (*P*<0.001), with post-hoc analysis revealing that food-seeking behaviour decreased for visits 2, 3, 4, and 5, compared with baseline (Fig 5). Further, food-seeking behaviour decreased significantly at visits 3, 4 and 5, compared with visit 2, and at visit 5 compared with visit 3.

## Discussion

This study is the largest international, multi-centre study conducted to date that that has examined controlled weight loss in overweight client-owned cats. The average weight lost was 10.6% SBW, and this equates to a mean rate of weight loss of 0.8% starting bodyweight per week. These outcomes are similar to those seen in a recent trial involving obese dogs (average weight loss 11.4% SBW; mean rate of weight loss 0.9% SBW per week) [30], but worse than most studies conducted in colony cats where rates of weight loss are typically 1–2% per week for a similar overall proportion of weight loss [9–17]. Further, unlike colony studies, many cats (41%) did not complete the trial, a proportion that is again similar to that seen in pet dogs (39%) [30]. This confirms findings from other studies that weight loss outcomes are broadly similar between pet dogs and cats [31–35] emphasising the fact that, although successful weight loss is possible, many pet cats and their owners will struggle. In the authors’ opinion, veterinary professionals should be advised to emphasise such challenges to owners when counselling them about what to expect during the weight loss period.

### Rate of weight loss

Rate of weight loss declined steadily throughout the 12-week period, even though attending veterinary professionals reduced food allocations when weight loss had slowed. Whilst this observation has been made in previous weight loss studies both in cats and dogs [20,30,36], the reason why it occurs is not clear. Possible reasons include the fact that energy expenditure might decline because of the energy restriction, or that owner compliance might worsen as the duration of weight loss increases. A decrease in mass-adjusted energy expenditure has previously been observed in cats during caloric restriction [37], and this would be consistent with the fact that a slowing of the rate of weight loss is sometimes observed in colony studies, where owner compliance is not a factor [9,17]. In contrast, in a recent colony study that used accelerometry and video to measure spontaneous physical activity, an increase in energy expenditure was observed during caloric restriction [14], a finding that is consistent with the results of the current study whereby owners subjectively judged the activity of their cats to have increased during weight loss. Regarding a possible association between poor owner compliance and the slowing rate of weight loss, owners feeding extra food despite veterinary recommendations to the contrary is common during periods of controlled weight loss in pet cats [19], likely because of the increased food-seeking behaviour observed with some weight loss food formulations [20]. Against this, most owners of cats in the current study did not observe an increase in food-seeking behaviour and, in fact, decreases in food-seeking behaviour were more commonly reported. These results contrast with findings from a recent study whereby owners reported increased appetite-related behaviours during weight loss in cats [38]. The reasons for the differences between studies are not clear, but might be the result of the switch to a high protein high fibre diet formulated for weight loss, known to reduce voluntary food consumption in cats compared with other diets [39]. Indeed, in the previous study, owners did not report differences in appetite-related behaviours between diet types (high fibre versus low carbohydrate), but the cats on the high fibre diet lost over twice as much weight, perhaps suggesting better compliance [38]. Nonetheless, these findings should be interpreted cautiously given that the owner observations were subjective and that the study did not include a comparator group (e.g. cats fed a different diet). Such observations might have been influenced by what veterinary professionals told owners about what to expect or about the characteristics of the weight loss diet (for example in promoting satiety); owners of cats who completed the trial might also have developed strategies to cope with food-seeking behaviours and thus did not feel it was a problem. Finally, since the trial was relatively short, it is unclear whether the apparent decline in food-seeking would have persisted, or could have worsened as weight loss progressed further. Therefore, further studies should be considered to determine how food-seeking behaviour changes during energy restriction whilst being fed a purpose-formulated weight loss diet.

### Effect of geographical location on outcome

A strength of the current study was its multicentre nature, in that cases were managed by over 100 practices from diverse geographical locations and, overall, weight loss was effective. That said, there were differences amongst geographical regions in the odds of a cat completing the 12-week trial. In this respect, cats in North America were more likely to complete than cats in Europe and Asia, whilst cats in Europe were more likely to complete the study than cats in Asia. These findings should be interpreted cautiously because the effect sizes were relatively small and confidence intervals were wide. Further, percentage weight loss was less in North American cats compared with those in Europe and South America. Similar observations were made when differences amongst countries were assessed, with some countries (e.g. USA) having a good completion rate but performing less well in terms of percentage weight loss, and other countries (e.g. Argentina, Italy, and UK) performing well in terms of percentage weight loss, but having a relatively poor completion rate. In most cases, this inverse association between completion rate and percentage weight occurred, and only one country (Germany) performed well both in terms of completion rate and percentage weight loss. Although the reasons for the difference in outcomes are not known, it might be related to the fact that compliance was better in the USA compared with elsewhere. It could be hypothesised that owners of cats that lose weight slowly are more likely to drop out, for example if they become frustrated with the lack of progress. Therefore, the better completion rate seen might indicate that veterinarians in the USA were better able to prevent cats losing weight slowly from dropping out, which improved compliance but at the expense of rate of weight loss and thus overall percentage of weight lost during the study. Other possibilities for the differences in outcome amongst different geographical locations and countries include differences in the characteristics of veterinarians, owners or cats recruited, or differences in support offered during the weight plan. One known difference in approach amongst countries was the method used for measuring food portion size: in the USA, calculated food portions were converted into a corresponding amount in terms of measuring cups whilst, in other countries, either electronic gram scales were used or a measuring cup was used which was marked with the correct portion size after first weighing a portion on electronic scales at the practice. The inaccuracy of measuring cups has been highlighted previously, whereby different people were asked to measure dry food portions of different size [40]. First, significant imprecision was noted with marked variability in the measurements made by different people, and variability remained even when the same person attempted to measure out the same portion size. Further, measurements were often inaccurate, with errors most frequently being made when measuring small portion sizes (i.e. amounts equating to those measured for cats), and most often being an over-estimate that could be up to 80% above actual portion size. Therefore, in the current study, use of measuring cups in the USA might have enabled owners to ‘cheat’ by giving their cats a few extra kibbles with each meal. It is notable that a similar difference in geographical location was seen in a recent global weight loss study involving dogs, although the effect was more marked in female compared with male dogs [30]. Although this difference might explain the poorer overall weight loss in cats from the USA, it is unclear why this did not affect overall completion. Future studies could consider comparing the effect of different methods of measurement on outcomes of weight loss in different geographic locations.

### Effect of sex and neuter status on outcome

In the current study, no effect of either sex on either completion rate or the percentage weight loss was observed. This is similar to findings from other studies in obese pet cats [19.20], but contrasts with the findings of a recent canine weight loss study, whereby female dogs lost more weight than male dogs [30]. This finding suggests that there is no need to adjust for sex when determining the starting energy allocation for weight loss in obese cats. No difference was also observed in outcomes for neutered versus sexually intact cats, again contrasting with recent work in dogs where sexually intact dogs lost more weight than neutered dogs [30]. Caution should be exercised when interpreting these findings because of the small number of sexually intact cats recruited, meaning that the study may have been underpowered to detect a small difference. Further studies targeting sexually intact cats would be needed to determine whether adjustments are needed when embarking on a weight loss plan.

### Effect of food type on outcome

An unexpected finding was the fact that, after multiple regression analysis, cats fed dry food exclusively were more likely to complete than those fed either wet food exclusively or fed a mix of wet and dry food. However, given the relatively small effect size (OR 0.69) and wide confidence intervals (95%-CI 0.48–0.95), this effect might not be clinically relevant and therefore should be interpreted cautiously. If a genuine effect, the result is intriguing given that dry food feeding has been identified as a risk factor for obesity in cats in one recent study [41], but not in many other studies [3,5,42–44]. To the authors’ knowledge, differing outcomes for cats fed wet and dry food have not been seen in previous feline weight loss studies. Nevertheless, dogs fed dry food were more likely to lose weight than those fed wet food or a combination of wet and dry food in a recent canine weight loss study [34]. Further, one previous feline weight loss study demonstrated that dietary energy intake had to be restricted more in cats fed wet food or a mix of wet and dry food, compared with those fed dry food exclusively, despite a similar rate of weight loss [19]. It is unclear why there might be a potential difference in outcome between cats on different diet formulations in the current study. One possible explanation relates to the method or portion allocation, in that dry food was most commonly measured out on electronic scales, meaning that portions could be accurately determined, and small adjustments to food allocation could be made when weight loss slowed. In contrast, wet food was fed either as whole or parts of a pouch or can, which might have led to inaccuracies in allocation. Further, it is easier to feed dry food using puzzle feeders, such as modified feeding bowls or feeding toys and, if such devices were used, they might have improved compliance. It is possible that the wet food used was significantly less palatable than the dry food, leading to more food refusals. Other possibilities include differences in palatability between the different diet formulations, or differences in the characteristics of cats who prefer wet and dry food, or differences in the owners that prefer to feed the different study types. That said, given the small effect size discussed above, further studies should be conducted both to confirm the finding and to determine the reason for it.

### Subjective changes in behaviour

As well as studying objective outcomes such as percentage weight lost, changes in activity and QOL were also assessed during the study. A significant improvement in activity was observed by visit 3 when an average of 5.5% SBW had been lost over a median of 33 days, and in QOL by visit 4 when an average of 8.1% SBW had been lost over a median of 61 days. These findings are similar to recent work in dogs that adopted the same approach, whereby improvements were seen within the first 2 weeks of weight loss [30], and also similar to studies in dogs that have used more objective measures of assessing mobility and QOL [45,46]. Of course, such results should be interpreted with caution because of the fact that the questionnaire had not been formally validated and also because there was no control group for comparison, for example that did not lose weight. Therefore, the changes might be in-part the result of a placebo effect invoked by starting a weight loss protocol and future studies should consider utilising more objective measures of activity and quality of life to confirm these findings. Nonetheless, the fact that owners have the impression of improvements is positive because it might help with compliance with future weight loss if owners believe that the weight loss is beneficial. Finally, the age of the cat also appeared to influence owner-reported activity and QOL were observed, with lower scores observed in the oldest cats. Whilst such effects might seem logical, they should again be interpreted with caution given the subject nature of these assessments.

### Limitations

The study has several limitations that should be acknowledged. First, it was only designed to be a short-term study meaning that most cats did not reach target weight by the final study visit. Therefore, it is unclear what overall success would have been had cats been followed longer, for example until they reached their target weight. That said, the fact that owners reported subjective improvements in both activity and QOL suggest improvements in health and welfare even though target weight was been reached. In fact, given the potential for failure with a complete weight loss programme, and the relatively high non-completion rate of the current study, ‘partial’ rather than complete weight loss programmes might be more appropriate when managing obese pet cats, as previously suggested [36]. Such a programme might invoke relative rapid health and welfare benefits, but be much more realistic for cat owners, especially those who find it difficult to comply. Even if such an approach were adopted, care should be taken to monitor weight after the period given the potential for weight regain to occur subsequently [47].

Second, no control group was included for comparison, meaning that similar results cannot necessarily be assumed if different foods are used for weight loss. Further, although a placebo effect is unlikely for the objective measurements (e.g. percentage weight loss), such an effect cannot be ruled out for the subjective measurements including changes in activity, QOL, and food-seeking behaviour. Nonetheless, that fact that owners ‘perceive’ improvements in behaviour even when these opinions are mistaken, is arguably a benefit since it might help compliance.

A third limitation was the fact that only 4 follow-up visits were formally scheduled and there was marked variability in the timings of these visits amongst cats. This might have had an adverse impact on the outcomes of weight loss that were observed, for example increasing non-completion and slowing weight loss. However, in many respects, this could be considered a study strength since this is likely to be the same as would happen with weight loss regimens in primary care practice. Fourth, to be eligible, cats had to be living in a single-cat indoor-only household. Whilst this helped to reduce study variability, it might have meant that the chance of a cat 'cheating' on its diet was less than would have been the case if multi-cat households and outdoor cats had been included. Further work is required to determine the impact of multi-cat households and outdoor access on weight loss in cats.

Finally, many cats were eligible for enrolment but the majority were either never enrolled or never completed the trial. Given the low recruitment and retention rate, results might not be generalisable to all obese cats. Nonetheless, the study was large and results were broadly consistent with those seen in other weight loss studies in overweight pet cats [17,18,21].

## Conclusion

In summary, we report the largest international multi-centre weight loss study ever conducted in overweight pet cats. Although most cats lost weight in all geographical locations, those in North America, and especially USA, lost less weight on average. Owners also reported subjective improvements in activity, QOL, commencing within the first month of weight loss, whilst food-seeking behaviour subjectively declined. These findings suggest that the same short-term weight loss protocol can be adapted to different geographical locations and settings.

## Supporting information

S1 Checklist Checklist for the STROBE statement. The table lists the items of the respective checklist, and the location within the manuscript where they can be found.
(DOC)

S1 Dataset Complete study dataset. Computer spreadsheet containing study data for the 710 cats in the study.
(XLSX)

S2 Dataset Dataset from the cats that completed the study. Computer spreadsheet containing study data for the subset of 413 cats completed the whole 5-visit study protocol.
(XLSX)
